# Functional Characterization of Serotonin N-Acetyltransferase Genes (*SNAT1*/*2*) in Melatonin Biosynthesis of *Hypericum perforatum*

**DOI:** 10.3389/fpls.2021.781717

**Published:** 2021-12-07

**Authors:** Wen Zhou, Shu Yang, Qian Zhang, Ruyi Xiao, Bin Li, Donghao Wang, Junfeng Niu, Shiqiang Wang, Zhezhi Wang

**Affiliations:** ^1^Key Laboratory of the Ministry of Education for Medicinal Resources and Natural Pharmaceutical Chemistry, National Engineering Laboratory for Resource Development of Endangered Chinese Crude Drugs in Northwest China, College of Life Sciences, Shaanxi Normal University, Xi’an, China; ^2^Shaanxi Engineering Research Centre for Conservation and Utilization of Botanical Resources, Xi’an Botanical Garden of Shaanxi Province, Institute of Botany of Shaanxi Province, Xi’an, China

**Keywords:** *Hypericum perforatum*, *Arabidopsis thaliana*, melatonin, SNAT gene family, serotonin N-acetyltransferase, hairy root

## Abstract

Hypericum perforatum is a traditional medicinal plant that contains various secondary metabolites. As an active component in *H. perforatum*, melatonin plays important role in plant antioxidation, growth, and photoperiod regulation. Serotonin N-acetyltransferase (SNAT) is the key enzyme involved in the last or penultimate step of phytomelatonin biosynthesis. A total of 48 members of SNAT family were screened and analyzed based on the whole genome data of *H. perforatum*, and two *SNAT* genes (*HpSNAT1* and *HpSNAT2*) were functionally verified to be involved in the biosynthesis of melatonin. It was found that *HpSNAT1* and *HpSNAT2* were highly expressed in the leaves and showed obvious responses to high salt and drought treatment. Subcellular localization analysis indicated that these two proteins were both localized in the chloroplasts by the *Arabidopsis* protoplasts transient transfection. Overexpression of *HpSNAT1* and *HpSNAT2* in *Arabidopsis* (SNAT) and *H. perforatum* (wild-type) resulted in melatonin content 1.9–2.2-fold and 2.5–4.2-fold higher than that in control groups, respectively. Meanwhile, *SNAT*-overexpressing *Arabidopsis* plants showed a stronger ability of root growth and scavenging endogenous reactive oxygen species. In this study, the complete transgenic plants of *H. perforatum* were obtained through *Agrobacterium*-mediated genetic transformation for the first time, which laid a significant foundation for further research on the function of key genes in *H. perforatum*.

## Introduction

*Hypericum perforatum*, commonly known as St. John’s wort (SJW), is an important medicinal plant used for centuries. Extracts of *H. perforatum* are widely used to treat anxiety, depression, sciatica, or even wounds because of their high efficiency, safety, and non-toxic side effects (Barnes et al., 2001). As an important bioactive component in *H. perforatum*, melatonin (N-acetyl-5-methoxytryptamine) is a ubiquitous multifunctional substance acting as a free radical scavenger, circadian rhythm regulator, and immune regulatory molecule (Galano et al., 2011; Manchester et al., 2015; Ma et al., 2020). It can induce antidepressant effects (Detanico et al., 2009), regulate some nerve regeneration processes (Atik et al., 2011), and protect organisms from environmental and internal oxidative stress (Galano and Reiter, 2018). Since discovering of melatonin in plants in 1995, many pharmacological studies have been carried out to explore its functional and physiological significance (Hattori et al., 1995; Tan et al., 2012; Hardeland, 2016). It has been reported that melatonin can not only regulate the whole growth and development stage of plants, from promoting seed germination to delaying leaf senescence; but also enhance the tolerance of plants to abiotic and biotic stresses, such as drought and salt stress, osmotic stress, extreme temperature, senescence, and pathogen attacks (Lee et al., 2014; Li et al., 2015; Liang et al., 2015; Han et al., 2017).

Melatonin is an indole heterocyclic compound synthesized from tryptophan which was confirmed by ^14^C-tryptophan isotope tracing experiment in *H. perforatum* (Murch et al., 2000). The biosynthesis of melatonin needs at least six enzymes after four consecutive enzymatic reactions, such as tryptophan decarboxylase (TDC), tryptamine 5-hydroxylase (T5H), tryptophan hydroxylase (TPH), serotonin N-acetyltransferase (SNAT), N-acetylserotonin O-methyltransferase (ASMT), and caffeic acid O-methyltransferase (COMT), among which SNAT is the penultimate enzyme or the last step enzyme involved in the melatonin biosynthesis. SNAT catalyzes 5-hydroxytryptamine (serotonin, 5-HT) to N-acetyl-5-hydroxytryptamine (NAS) or 5-methoxytryptamine (5-MT) to melatonin, and the subcellular localization analyses of SNAT in different plants was almost located in chloroplasts (Lee et al., 2015a; Byeon et al., 2016). Since the first plant SNAT gene was identified and cloned in rice, it was found that the genes belong to GCN5 related N-acetyltransferase (GNAT) superfamily playing a critical role in regulating the accumulation of melatonin and were located in chloroplasts (Byeon et al., 2016; Lee and Back, 2017). Subsequently, Lee et al. (2015b) cloned *AtSNAT* from *Arabidopsis* and the results showed that the melatonin content of the gene knockout mutant decreased significantly and was more susceptible to *Pseudomonas syringae* infection (Byeon et al., 2015a). Wang et al. overexpressed apple *MzSNAT5* in *Arabidopsis* wild-type plants, which increased the content of melatonin in *Arabidopsis* mitochondria by about three times, thus enhancing the drought resistance of transgenic plants (Lin et al., 2017). Park et al. reported that *PtSNAT* cloned from *Pinus taeda* was also located in chloroplasts (Sangkyu et al., 2014). Byeon et al. revealed that the melatonin content in the *OsSNAT* (AK059369) RNAi line was significantly lower than that in the wild-type plants, and the seedlings grew slowly and had weak tolerance to CdCl_2_ stress (Byeon and Back, 2016). Wang et al. (2020) found that compared with wild-type plants, the expression of *SlSNAT* (Solyc10g074910) in overexpressed lines increased by about 3–5 times, and the content of melatonin increased by about 3–4 times. The homologous *SNAT* genes in other species such as cyanobacteria (Byeon et al., 2013b), *Vitis vinifera* (Yu et al., 2019), and *Pyropia yezoensis* (Byeon et al., 2015b) have also been identified and characterized by enzymology.

Regulating the expression of enzyme genes or transcription factors through metabolic engineering can effectively improve the biosynthesis of therapeutic compounds in medicinal plants (Rathinasabapathi, 2000; Capell and Christou, 2004). Although DNA recombinant technology has been widely used, genetic improvement in plants is still challenging without robust transformation methods. In recent years, it has been found that the resistance of *H. perforatum* to *Agrobacterium tumefaciens*-mediated leaf disk transformation is largely due to the induction of plant defense response (Hou et al., 2016). Through co-culture with *Agrobacterium rhizogenes*, some research groups obtained positive *H. perforatum* transgenic hairy roots (HR) (Koperdáková et al., 2009; Tusevski et al., 2012; Montazeri et al., 2019). However, *H. perforatum*, the whole grass is used as medicine. If complete transgenic plants are not obtained, the expression detection of many target genes will be limited. Therefore, *A. rhizogenes* was used to infect *H. perforatum* tissue in this study. By optimizing the conditions of *Agrobacterium* infection and explant growth, HRs, and completely transformed plants were successfully induced. The genetic transformation system of *A. rhizogenes* was established.

In this study, based on the whole-genome sequence of *H. perforatum*, the HpSNAT gene family was screened. The key genes *HpSNAT1* and *HpSNAT2* were identified and functionally validated in *Arabidopsis* and *H. perforatum*. Second, the regeneration of mature transgenic plants from transformed *H. perforatum* root cultures was successfully achieved.

## Materials and Methods

### Identification and Sequence Analysis

To identify the *HpSNAT* candidates, the hidden Markov model profile of the SNAT conserved DNA binding domain (PF13508) was used as a query to search the genomic databases of *H*. *perforatum via* the PFAM databases.^1^ In addition, only those genes with complete GNAT domain can be used as members of the SNAT gene family by using InterProScan^2^ (Zdobnov and Rolf, 2001). The isoelectric point (PI) and molecular weight (MW) of the HpSNAT proteins were predicted by Compute PI/MW tool on the ExPASy^3^ (Gasteiger, 2005).

### Isolation and Bioinformatics Analysis of *HpSNAT1* and *HpSNAT2*

Four *SNAT* genes (rice *OsSNAT1* and *OsSNAT2*, *Arabidopsis AtSNAT1* and *AtSNAT2*) were confirmed to have SNAT activity, identified as bait proteins. A neighbor-joining phylogenetic tree of the candidate HpSNATs and four bait protein sequences was constructed using the software MEGA 9.0 with 1000 replicates bootstrap. The protein sequence of HpSNATs was used to predict the conserved motifs by using the program MEME^4^ (Bailey et al., 2009) with the following parameters: a maximum number of motifs sets at 5, optimum motif width from 70 to 300 bp, and with an *e*-value less than 1e-10. The amino acid sequence of the conserved GNAT domain of HpSNAT proteins was obtained by SMART,^5^ and then multiple sequence alignment analysis was carried out by using Geneious v10.22. The upstream 2 kb genomic DNA sequences of *HpSNAT1*/*2* were uploaded to the PlantCARE database to search for the *cis*-acting regulatory elements in the promoter region (Lescot, 2002). The transcriptome data of different tissues (root, stem, leaf, and flower) were used to display the tissue-specific expression profile in the form of a heat map by using Origin v10.5.

### Plant Material and Stress Treatment

*Arabidopsis* homozygous mutant *snat* (SALK_033944C) and wild-type Columbia-0 (Col-0) seeds were obtained from the European *Arabidopsis* Stock Centre (NASC). Three primers (LP, RP, and BP) were used to screen the homozygous mutants (Supplementary Figure 1 and Supplementary Table 1). The seeds of *H*. *perforatum* were preserved in our laboratory. The sowing, sterilization, and growth conditions of aseptic tissue culture seedlings of *Arabidopsis* and *H*. *perforatum* can be referred to in the previous detailed description (Zhou et al., 2021). Three-month-old aseptic seedlings of *H*. *perforatum* were transferred into liquid Murashige and Skoog (MS; Solarbio) medium containing 250 mM D-Mannitol (Alfa Aesar) and 200 mM NaCl (Solarbio) for drought and high salt stress treatment. One-week-old aseptic seedlings of *Arabidopsis* were transferred to one-half strength MS agar medium with 200 mM D-mannitol and 150 mM NaCl for 7 days to observe their phenotypes, while 4-week-old seedlings in soil were treated with drought stress without watering for 12 days.

### Gene Cloning and Vector Construction

Scaf 151.204 (*HpSNAT1*) and Scaf 64.495 (*HpSNAT2*) were cloned from *H*. *perforatum* transcriptome cDNA (Zhou et al., 2021) using gene-specific primers (Supplementary Table 1). The full-length cDNAs mentioned above were inserted into PBI221-GFP to produce the *35S*::*SNAT*-*GFP* constructs as subcellular localization vectors. The *HpSNAT* genes were cloned with the Gateway™ Recombination Cloning Technology (Invitrogen, Waltham, MA, United States) into the pEarleyGate202 vector to generate final overexpression (OE) vectors pEG202-*HpSNAT1* and pEG202-*HpSNAT2*.

### Genetic Transformation and Growth Conditions

For overexpressing *HpSNAT* in *Arabidopsis*, the pEG202-*HpSNAT* constructs were introduced into *Agrobacterium tumefaciens* strain GV3101 (self-made) and transformed into *Arabidopsis snat* plants by the floral dip method (Zhang et al., 2006). According to the resistance of the expression vector to BASTA, the positive transgenic plants were screened out until T3 generation (Supplementary Figure 2).

For overexpressing *HpSNAT* in *H*. *perforatum*, the recombinant constructs were transferred into *Agrobacterium rhizogenes* strain K599 (Weidi, Shanghai, China). The specific steps follow the product instructions. A culture of *A. rhizogenes* with pEG202- *HpSNAT1* and pEG202-*HpSNAT2* was grown for 1 day at 28°C and 120 rpm in TY liquid media (0.5% tryptone, 0.3% yeast extract, 0.5% meat extract, 10 mM CaCl_2_, 1.5% agar with 50 μg/ml kanamycin, and 50 μg/ml streptomycin; pH 7.0). The bacterial suspension was centrifuged at 5,000 rpm for 10 min and resuspended in sterile water, then diluted to an OD_600_ of 0.1.

#### Hairy Root Induction

The roots of 3-month-old aseptic seedlings used as explants were cut into 1.0–1.5 cm segments and placed on solid B5 medium (Solarbio; supplemented with 3% sucrose, 0.7% agar, and pH 5.8) without antibiotics in an incubator for 2 days (25°C, dark). Then the pre-cultured segments were put into the diluted bacterial solution for transfection at 25°C. During this period, the tube was slightly reversed several times to make the roots fully immersed. After 15 min, the root segments were taken out to absorb the remaining surface solution and placed on solid B5 medium for 2 days (25°C, dark). Afterward, the transfected explants were transferred to solid B5 medium containing 200 μg/ml cefotaxime and 2 μg/ml BASTA (25°C, dark) for transgenic explants selection.

#### Hairy Root Propagation

When growing to 6 weeks, samples of the transgenic roots weighing 100 mg were inoculated into 250 ml beaker flasks containing 100 ml liquid MS medium (supplemented with 0.2 μg/ml naphthyl acetic acid and 50 μg/ml cefotaxime) and then placed on an orbital shaker at 110 rpm/min, 25°C in the dark. After 1 week, the medium was changed to liquid MS medium only supplemented with 50 μg/ml cefotaxime, which were regularly sub-cultured (every 1 week). These lines cultured for 2 months were used for DNA and RNA analysis. The positive transgenic lines were detected by amplifying the CaMV35S promoter sequence.

#### Calli Induction and Plant Regeneration

The positive and high expression of HR were cut into 1.0–1.5 cm root segments and put on solid MS medium (supplemented with 0.1 μg/ml thidiazuron and 50 μg/ml cefotaxime) for callus induction (25°C, dark). Two months later, the green calli were cut off and placed on solid MS medium (supplemented with 1 μg/ml 6-Benzylaminopurine and 0.1 μg/ml naphthylacetic acid) maintained at 25°C with a 16 h photoperiod at 108 μmol/m^2^/s for the induction of adventitious buds.

#### Root Induction

When the adventitious buds grew to about 2 cm, they were cut off and placed on one-half strength solid MS medium without phytohormones for roots induction at the same growth conditions. The above-mentioned medium was changed every 2 weeks. After 4–5 months, the aboveground part of the seedlings was used to detect the expression patterns of *HpSNAT* and the content of the metabolites.

### PCR Analysis and Subcellular Localization Analysis

Genomic DNA and RNA were extracted using the FastPure Plant Total RNA/DNA Isolation Kit (Vazyme, Jiangsu, China). The specific extraction steps are by the product instructions. The CaMV35S promoter was amplified from the genomic DNA to detect whether the gene was integrated into the genome of the transgenic plants. PCR amplification was performed as previously described (Wang et al., 2018). Reverse transcription PCR (RT-PCR) was performed to explore the expression patterns of *HpSNAT* genes in *Arabidopsis snat*, Col-0, and T3 transgenic plants. An *Arabidopsis Actin* gene fragment is used as an internal control. Quantitative real-time PCR (qPCR) was used to investigate the expression patterns of *HpSNAT* genes in *H*. *perforatum* plant, and the *HpACT2* gene was used as an internal reference. The specific operation steps can be referred to in the previous detailed description (Zhou et al., 2021) and primer sequences used are shown in Supplementary Table 1. The transient transformation of *Arabidopsis* protoplasts is operated according to Dr. Sheen’s operation manual^6^ (Sheen, 2001). Briefly, the leaves of *Arabidopsis* plants aged 3–4 weeks were cut into 1 mm wide bands, completely soaked in 10 ml enzymatic hydrolysate and digested in the dark at 55°C water bath for 10 min. Then, digestion was continued at 25°C for about 5 h and gently shaken in the dark. The samples were washed with solution W5 and filtered through a 300-mesh sieve. The obtained protoplasts were resuspended in MMG solution for microscopic examination. After PEG-mediated transfection, the fluorescence was observed by Nikon C2-ER laser confocal microscope.

### Measurement of Melatonin and Its Precursors

Two-month-old *Arabidopsis* OE, Col-0, and *snat* plants were collected. According to the extraction and detection methods established in our laboratory, melatonin in *Arabidopsis* leaves was determined by LC/MS (Zhou et al., 2021). The leaves of OE and WT (the line infected by *A*. *rhizogenes*, K599 only) lines of *H*. *perforatum* were freeze-dried, and a 200 mg dry sample was extracted by ultrasonic treatment in 500 μl 80% methanol (Sigma, St. Louis, MO, United States) (Zuo et al., 2015). The sample preparation and HPLC detection of melatonin and its precursors were performed as previous described (Zhao et al., 2013).

### Physiological Assays

One-week-old OE, Col-0, and *snat* aseptic seedlings of *Arabidopsis* under drought and high salt stress were to observe their phenotypes. One-month-old plants without watering to create drought stress for physiological analysis. The concentration of H_2_O_2_ and Malondialdehyde (MDA) were measured using the Hydroperoxide/Malondialdehyde Fluorometric Assay Kit (Sigma–Aldrich). Reactive oxygen species (ROS) concentration was examined by histochemical staining with CM-H_2_DCFDA fluorescent dye (Solarbio, Tongzhou District, Beijing, China) (Oparka et al., 2016). Fluorescence images were taken with a Leica stereomicroscope (Leica, Germany).

### Statistics

All experiments were performed with three biological replicates unless otherwise specified. The data were the average of three technical repetitions expressed as mean ± standard error. ANOVA analysis was used for statistical analysis; the probability value *P* < 0.05 was considered statistically significant.

## Results

### Isolation and Bioinformatics Analysis of *Hypericum perforatum* Serotonin N-Acetyltransferase

**FIGURE 1:**
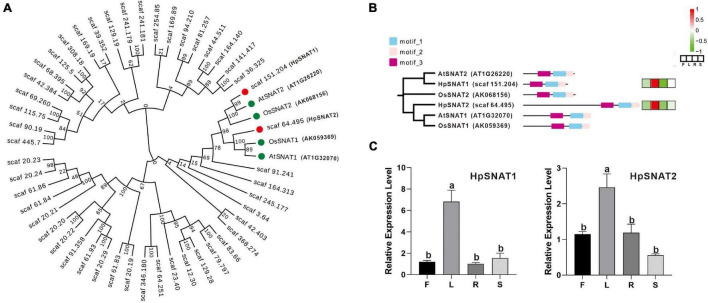
Bioinformatics analysis of HpSNAT. **(A)** The phylogenetic tree of HpSNAT and the reference proteins AtSNAT1, AtSNAT2, OsSNAT1, and OsSNAT2 from *Arabidopsis thaliana* and *Oryza sativa*. The sequences were analyzed by MEGA 10.0 using bootstrap analysis with 1000 replicates. **(B)** The distribution of three conserved motifs and heat maps of six SNAT proteins. The heatmaps were analyzed by the FPKM values (F: flowers, L: leaves, R: roots, and S: stems) for each protein (*Z*-score). **(C)** Relative quantitative analysis of *HpSNAT1* and *HpSNAT2* in different tissues of *Hypericum perforatum*. Data were normalized by *HpACT2* (MK054303), calculated with the equation 2^−△△Ct^. All data represent averages of three biological replicates, error bars indicate ± SD. Different letters indicate significant differences from the control (*P* < 0.05) tested by one-way ANOVA.

A total of 48 HpSNAT members were screened, all of which contained GNAT conserved domain. The MW of the predicted HpSNAT proteins ranged from 14.92 kDa (HpSNAT26) to 132.67 kDa (HpSNAT21), the PIs ranged from 4.98 (HpSNAT23) to 9.97 (HpSNAT1), and protein lengths ranged from 129 (HpSNAT26) to 1,178 (HpSNAT21) amino acids (Supplementary Table 2). Hierarchical clustering of expression profiles of HpSNAT genes in different tissues (root, stem, leaf, and flower) were shown in Supplementary Figure 3. Among them, two full-length HpSNAT showed homology to the four bait proteins, and they were named *HpSNAT1* (Scaf 151.204) and *HpSNAT2* (Scaf 64.495) according to the phylogenetic analysis (Figure 1A). As shown in Figure 1B, two HpSNATs and four bait proteins have the same motif, showing that the functional proteins have similarities. The qPCR results were consistent with the expression pattern of *HpSNAT1* and *HpSNAT2* in different tissue transcriptome sequencing results, indicating that the two genes were highly expressed in leaves (Figure 1C). Through the analysis of *cis*-acting elements in the promoter region of the two genes, it was found that both two genes contain gibberellic acid (GA), methyl jasmonate (MeJA), salicylic acid (SA), auxin (IAA), abscisic acid (ABA), and stress response elements of low temperature, drought, and injury. In addition, *HpSNAT1* also contains flavonoids biosynthesis and elements related to plant circadian rhythm regulation, while *HpSNAT2* also contains ethylene response and elements related to gene expression regulation in plant meristem.

### Subcellular Localization of HpSNAT

**FIGURE 2:**
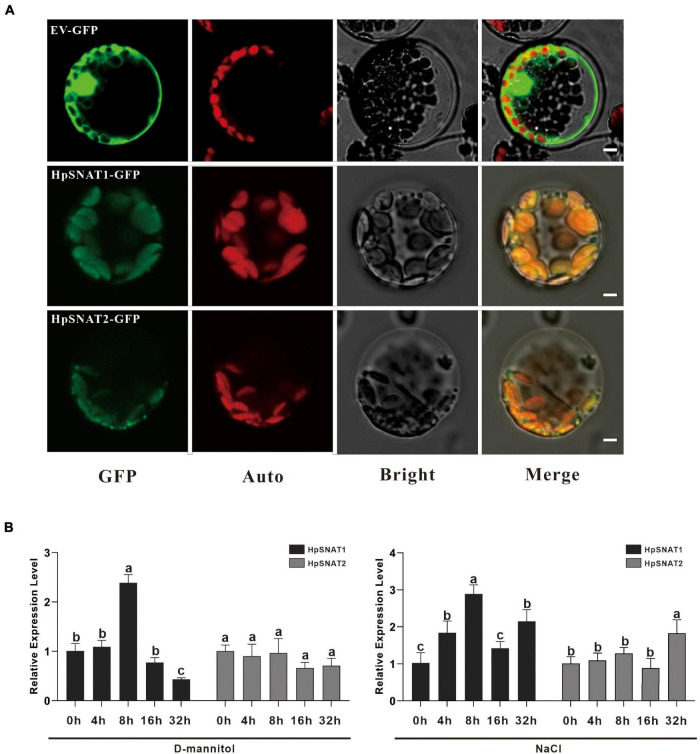
Subcellular localization and expression patterns of HpSNAT. **(A)** From left to right are the subcellular localization of the target genes (GFP), chloroplast autofluorescence (Auto), bright-field (Bright), and Merged (Merge) images. Scale bars = 5 μm. **(B)** Relative quantitative analysis of *HpSNAT1* and *HpSNAT2* under drought (D-mannitol) and high-salt (NaCl) stress conditions. Data were normalized by *HpACT2* (MK054303), calculated with the equation 2^−△△Ct^. All data represent averages of three biological replicates, error bars indicate ± SD. Different letters indicate significant differences from the control (*P* < 0.05) tested by one-way ANOVA.

To determine the subcellular location of HpSNAT1 and HpSNAT2 *in vivo*, HpSNAT-GFP fusion proteins expression vector driven by the CaMV 35S promoter was constructed, and the empty vector PBI221-GFP was used as a positive control. The fused expression vector was used to perform a transient expression assay in *Arabidopsis* protoplasts. From Figure 2A, the GFP fluorescence of positive control was almost distributed in the whole cell. In contrast, the GFP fluorescence of HpSNAT1/2 was observed only in the chloroplasts in cytoplasm, which was consistent with the previous subcellular localization of SNAT proteins in other plants (Byeon et al., 2013a; Lee et al., 2015a; Yeong et al., 2016).

### Expression Patterns of HpSNAT

Melatonin has strong free radical scavenging and antioxidant ability to prevent cell oxidative damage. To determine the effects of drought and high salt stress on the expression of *HpSNAT1* and *HpSNAT2*, 3-month-old aseptic seedlings of *H*. *perforatum* were treated with 250 mM D-Mannitol and 200 mM NaCl for up to 32 h. The expression trend of the *HpSNAT1* and *HpSNAT2* in *H*. *perforatum* at five-time intervals (0, 4, 8, 16, and 32 h) was quantified by qPCR (Figure 2B). Under drought stress, the expression level of *HpSNAT1* reached the highest at 8 h, increased by about 2.5 times, while *HpSNAT2* had no obvious response to D-mannitol treatment. Similarly, the expression level of *HpSNAT1* increased nearly three-fold at 8 h under high salt stress, while *HpSNAT2* showed a slight response at 32 h.

### Overexpression of HpSNAT Increased Melatonin Accumulation in *Arabidopsis*

**FIGURE 3:**
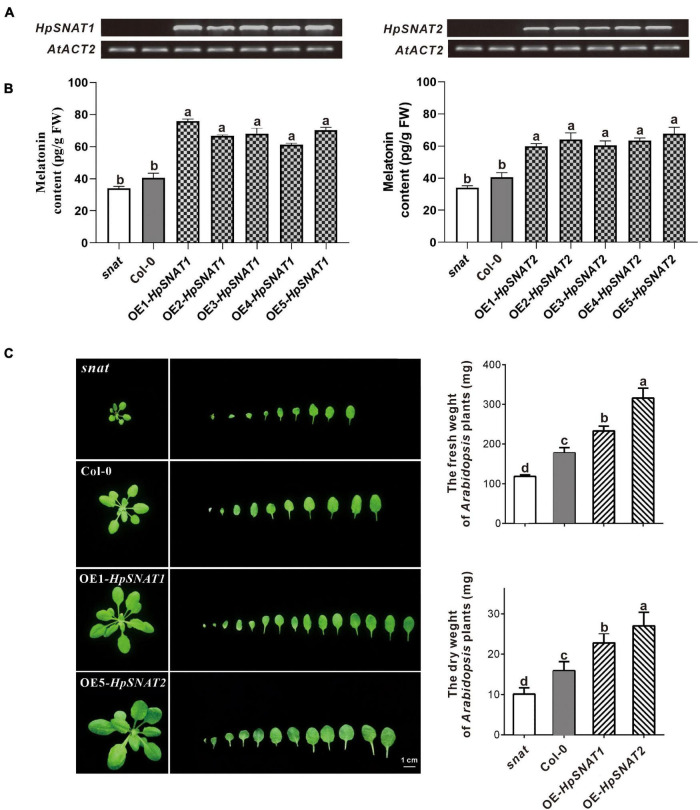
The melatonin content and phenotype of *snat*, Col-0, and overexpressed *Arabidopsis* (OE). **(A)** and **(B)** Expression levels of HpSNAT and melatonin content in *snat*, Col-0, and five OE lines. HpSNAT expression was analyzed by RT-PCR. **(C)** Comparison of the rosette leaves in *snat*, Col-0, and OE lines. Each value represents the average of three replicates, and the error bars represent ± SD. Different letters indicate significant differences from the control (*P* < 0.05) tested by one-way ANOVA.

By overexpression of *HpSNAT1* and *HpSNAT2* in the *Arabidopsis* mutant *snat*, the OE lines contained the expected 926 bp fragments of the CaMV35S promoter were confirmed by PCR. Five T3 homozygous transgenic lines of OE-*HpSNAT1* and OE-*HpSNAT2* were obtained for gene expression (Figure 3A) and melatonin detection (Figure 3B). Finally, three OE lines with high *HpSNAT1* (OE1, OE3, and OE5) and *HpSNAT2* (OE2, OE4, and OE5) gene expression and melatonin content were used for further analysis. The content of melatonin in OE1- *HpSNAT1* was 1.86 times higher than that in Col-0 plants, and that in OE5-*HpSNAT2* was 1.67 times higher. By analyzing the rosette leaves of different lines (*snat*, Col-0, OE1- *HpSNAT1*, and OE5-*HpSNAT2*) growing to 25 days, we found that the OE lines had more leaves than *snat* and Col-0. The dry and fresh weight of OE leaves was almost three times that of the *snat* and twice that of Col-0 (Figure 3C).

### Overexpression of HpSNAT Enhanced Drought and Salt Tolerance in *Arabidopsis*

**FIGURE 4:**
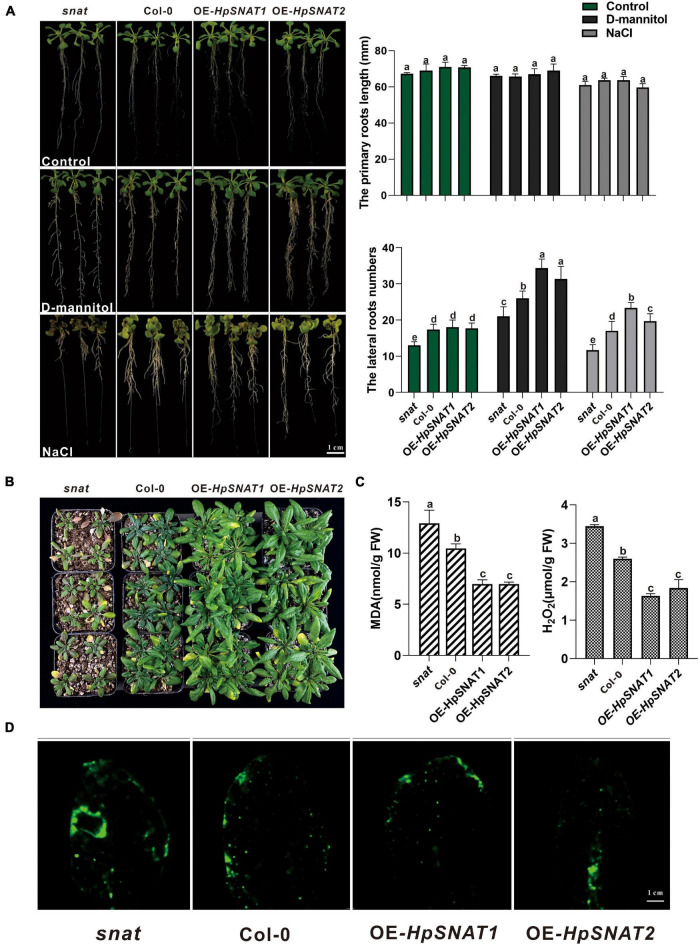
Function of *HpSNAT1* and *HpSNAT2* when expressed in *Arabidopsis* plants. **(A)** The phenotype, primary root length, and lateral root number of *snat*, Col-0, and OE seedlings under normal condition (control), D-mannitol, and NaCl treatments. Each value represents the average of three replicates, and the error bars represent ± SD. Different letters indicate significant differences from the control (*P* < 0.05) tested by one-way ANOVA. **(B)** Drought tolerance test. Four-week-old plants of *snat*, Col-0, and OE in soil were dehydrated for 12 days. **(C)** MDA and H_2_O_2_ accumulation in *snat*, Col-0, and OE lines under drought stress. Each value represents the average of three replicates, and the error bars represent ± SD. Different letters indicate significant differences from the control (*P* < 0.05) tested by one-way ANOVA. **(D)** Histochemical staining with fluorescent CM-H2DCFDA to detect reactive oxygen species in *snat*, Col-0, and OE lines under drought stress.

In order to explore whether *HpSNAT* genes can enhance the drought and salt resistance of plants, *Arabidopsis* root morphology of different lines was observed in one-half-strength MS medium with NaCl and D-mannitol, respectively. Results as shown in Figure 4A, after 10 days of treatment, there was no significant change in the length of the primary root of *snat*, Col-0, and OE, but the number of lateral roots of OE lines was significantly more than that of Col-0 and *snat* plants under drought and salt stress. Similarly, the growth of 4-week-old seedlings in soil was better than that of Col-0 and *snat* after without watering for 12 days (Figure 4B), and the MDA and H_2_O_2_ contents in OE lines were significantly lower than in Col-0 and *snat* plants (Figure 4C). From the CM-H_2_DCFDA staining, it can be seen that the fluorescence intensity in OE leaves (Figure 4D) is lower than that in *snat* under drought treatment. The relative staining fluorescence intensities in *snat*, Col-0, OE-*HpSNAT1*, and OE-*HpSNAT2* leaves were 91.455, 71.502, 63.009, and 60.531, respectively. These results showed that the content of ROS in transgenic leaves was lower than that in Col-0 and *snat*. In other words, the content of ROS in plants can be greatly reduced by overexpression of *HpSNAT* genes, so as to improve the drought tolerance of plants.

### Hairy Root Induction in *Hypericum perforatum*

**FIGURE 5:**
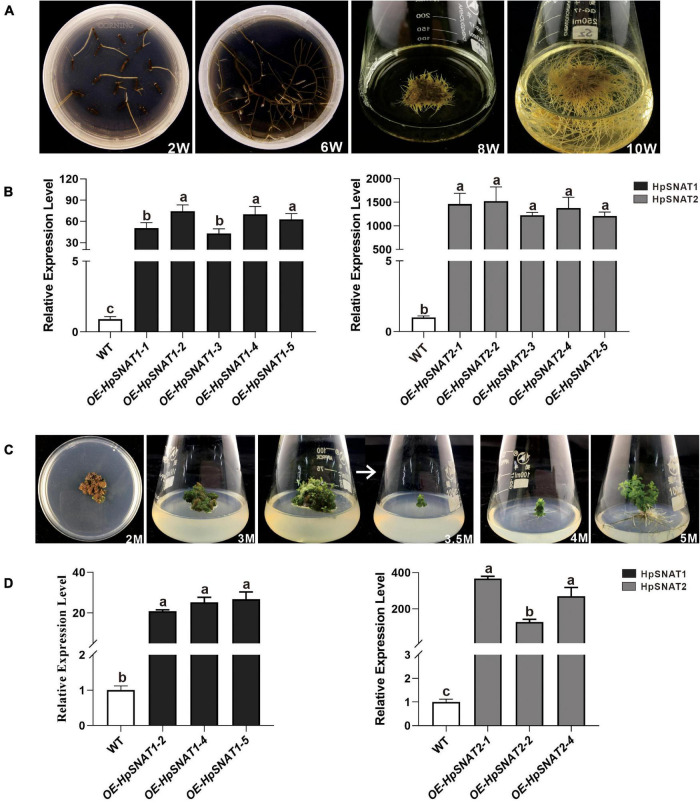
Selection of positive hairy roots (HRs) lines and generation of adult transgenic *H. perforatum*. **(A)** The phenotypes of HRs induction at different time (W: week). **(B)** Relative quantitative analysis of *HpSNAT1* and *HpSNAT2* expression in overexpression (OE) and control of *H. perforatum* HRs (WT: the line infected by *A*. *rhizogenes*, K599 only). Data were normalized by *HpACT2* (MK054303), calculated with the equation 2^−△△Ct^. All data represent averages of three biological replicates, error bars indicate ± SD. **(C)** Plant regeneration from *H. perforatum* HR cultures (M: month). **(D)** Relative quantitative analysis of *HpSNAT1* and *HpSNAT2* expression in OE and control of *H. perforatum* plants. Data were normalized by *HpACT2* (MK054303), calculated with the equation 2^−△△Ct^. All data represent averages of three biological replicates, error bars indicate ± SD.

As shown in Figure 5A, the first candidate transgenic root tips emerged from the wounded areas of the explants about the 2nd week on the selective medium with antibiotics. When growing to the 6th week, 100 mg roots were put into a liquid MS medium containing phytohormones for expanded culture to obtain enough samples for molecular detection. After 10 weeks of growth, the positive lines carrying the *HpSNAT* gene were verified by PCR. In total, we obtained five positive transgenic HR lines for both constructs that were confirmed in *HpSNAT* gene expression. The expression levels of the two genes in the OE lines were significantly higher than those in the WT group, especially the OE2, OE4, and OE5 of *HpSNAT1* and OE1, OE2, and OE4 of *HpSNAT2* detected by qPCR (Figure 5B). Among them, the expression of *HpSNAT2* in the OE lines increased by about 1500 times. The above highly expressed HR lines were selected for callus, adventitious bud, and root induction to obtain a complete transgenic plant of *H*. *perforatum*.

### Transgenic Lines Selection in *Hypericum perforatum*

**FIGURE 6:**
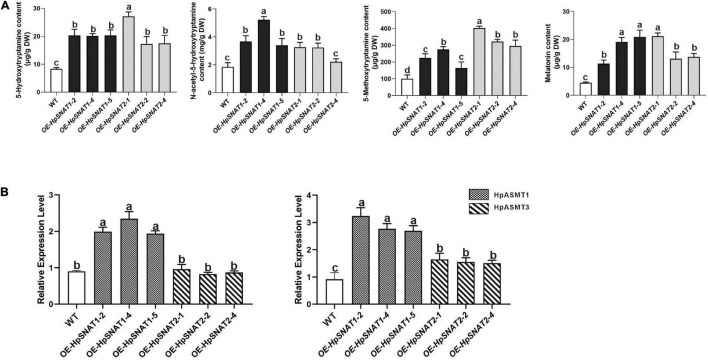
Secondary metabolites contents and expression patterns of *HpASMT* in *H. perforatum*. **(A)** Comparisons of 5-HT, 5-MT, NAS, and melatonin in OE and control of *H. perforatum* plants. **(B)** Relative quantitative analysis of *HpASMT1* and *HpASMT3* in transgenic lines and control of *H. perforatum* plants. Data were normalized by *HpACT2* (MK054303), calculated with the equation 2^−△△Ct^. All data represent averages of three biological replicates, error bars indicate ± SD.

As shown in Figure 5C, when explants were grown on MS medium containing thidiazuron for 2 months, a large amount of brown callus will be formed with a little green part in it. Then the green callus was separated and transferred to MS medium containing 6-Benzylaminopurine and naphthylacetic acid to induce adventitious buds. About 1 month later, the shoots were cut off and inserted into one-half strength MS medium for rooting induction to obtain complete plants. It began to take root slowly in about 2 weeks, and lots of HRs and branches could be found outside the medium after 1 month. qPCR analysis of HpSNAT expression showed that *HpSNAT1* expressed over 20-fold higher than the WT transformed lines, and the expression of *HpSNAT2* was 100- to 400-fold higher in adult transgenic *H*. *perforatum* lines (Figure 5D). We further evaluate the content of melatonin and its precursors. Three OE and WT lines were used to examine four compounds, such as 5-HT, NAS, 5-MT, and melatonin in adult transgenic *H*. *perforatum*. The results showed that the contents of four compounds were significantly up-regulated, and the melatonin increased to 12.28–21.15 μg/g DW in OE lines (Figure 6A). Among them, OE-*HpSNAT2*-1 accumulated the highest content of melatonin, which was 4.21-folds higher than WT. These results confirmed the positive role of *HpSNAT1* and *HpSNAT2* in melatonin biosynthesis.

## Discussion

In the past 20 years, the research of phytomelatonin mostly focused on the content detection in the early stage. However, due to its diverse and important physiological functions, melatonin soon attracted extensive attention in the research of herbal medicine, horticultural plants, and crops. Although great progress has been made in the research of phytomelatonin, there are still many problems to be clarified compared with plant hormones such as auxin, abscisic acid, ethylene, polyamine, and brassinolide. Many studies have proved that applying of a certain concentration of exogenous melatonin could enhance the resistance of plants to the extreme environments and improve the disease resistance of plants (Li et al., 2012; Yin et al., 2013; Xian et al., 2018; Lin et al., 2019). Therefore, how to improve the content of endogenous phytomelatonin through modern breeding methods to improve stress resistance will be the key research field of using phytomelatonin to improve yield and product quality in the future. SNAT is a key rate-limiting enzyme in melatonin synthesis and plays an important role in regulating the balance of melatonin accumulation (Lee et al., 2015b; Yeong et al., 2015). The related research of the SNAT gene family mainly focuses on the model plants *Arabidopsis* and rice, while there is a scarce study on the medicinal plants such as *H*. *perforatum*. Therefore, it is necessary to expand the research object for the in-depth exploration and interpretation of many physiological processes.

In this study, 48 SNAT genes were screened based on the *H*. *perforatum* whole-genome database. There was high homology of amino acid sequence between the HpSNAT and AtSNAT/OsSNAT (homology from 42.34 to 70.59%). Phylogenetic analysis was also consistent with amino acid homology analysis, indicating that *HpSNAT* may have similar gene functions with *AtSNAT* and *OsSNAT*. In order to preliminarily explore the possible functions of *HpSNAT1* and *HpSNAT2*, we measured their subcellular localization and expression patterns in different tissues and under different stress. The two proteins were both located on the chloroplast, and the high expression of *HpSNAT1* and *HpSNAT2* in leaves also complements the results of subcellular localization. Chloroplast is the place of photosynthesis and also produces many free radicals at the same time. The production of melatonin in chloroplasts can be explained from an evolutionary point of view (Tan et al., 2013).

Increasing the content of bioactive compounds is the main aim of *H*. *perforatum* genetic engineering. Although the overexpression of genes involved in biosynthesis will enable us to achieve the above objectives, the pathway engineering of this species is still in the primary stage, mainly due to the lack of genetic information about these biosynthetic pathways and effective transformation methods. By overexpressing *HpSNAT1* and *HpSNAT2* in *Arabidopsis* mutant *snat*, we observed that the melatonin level in OE lines was nearly two times higher than that of Col-0 and mutant plants. When plants are under stress, the balance of the active oxygen metabolism system is broken, and the production of ROS increases. Accordingly, transgenic plants also showed stronger drought and salt tolerance compared with Col-0 and *snat*. These results indicate that overexpression of *HpSNAT* genes in *Arabidopsis* improves the ability of plants to resist adverse environments, which is directly related to the strong antioxidant function of melatonin. Although *A*. *tumefaciens*-mediated transformation of *H*. *perforatum* has not been reported, the study of inducing HRs after co-culture with *A*. *rhizogenes* has been comparatively mature. In this study, when the HRs of positive transgenic *H*. *perforatum* were successfully obtained, complete transgenic plants were formed through dedifferentiation and redifferentiation induced by plant hormones. Finally, a stable and efficient genetic transformation system of *H*. *perforatum* was successfully established, which solved the problem of restricting the genetic transformation of *H*. *perforatum*. The contents of melatonin and its precursors (5-HT, 5-MT, and NAS) in different lines were detected. The results showed that the contents of these substances in OE lines were significantly higher than those in WT plants. It is well known that SNAT protein converted 5-HT and 5-MT into NAS and melatonin, while ASMT protein converted 5-HT and NAS into 5-MT and melatonin. Therefore, we further detected *HpASMT1* and *HpASMT3* in the OE lines and control group, and the results showed that the expression of *HpASMT* genes was also obviously up-regulated (Figure 6B). It indicated that the overexpression of *HpSNAT1* and *HpSNAT2* would not only increase the content of NAS and melatonin in *H*. *perforatum*, but also up-regulate the expression of *HpASMT1* and *HpASMT3* in the melatonin metabolic pathway to increase the content of melatonin and other precursors. It is also confirmed that these enzyme genes in the melatonin biosynthesis pathway have synergistic regulation (Byeon et al., 2015c).

## Conclusion

This study defines a foundation for identifying and functional characterization of the role of SNAT genes in the species *H*. *perforatum*. It not only clarified the role of *HpSNAT* genes in phytomelatonin biosynthesis, but also provided an important basis for the molecular mechanism of melatonin regulating plant growth and stress response. In addition, the successful establishment of Agrobacterium-mediated transformation system of *H*. *perforatum* lays a foundation for the follow-up study of key genes functions.

## Data Availability Statement

The datasets presented in this study can be found in online repositories. The names of the repository/repositories and accession number(s) can be found below: https://www.ncbi.nlm.nih.gov/, PRJNA588586.

## Author Contributions

WZ and ZW designed the study. SY, WZ, and QZ performed the experiments and analyzed the data. RX, DW, SW, and JN contributed analytical tools and provided technical support. WZ wrote the first draft. ZW approved the final draft of the manuscript. All authors contributed to the article and approved the submitted version.

## Conflict of Interest

The authors declare that the research was conducted in the absence of any commercial or financial relationships that could be construed as a potential conflict of interest.

## Publisher’s Note

All claims expressed in this article are solely those of the authors and do not necessarily represent those of their affiliated organizations, or those of the publisher, the editors and the reviewers. Any product that may be evaluated in this article, or claim that may be made by its manufacturer, is not guaranteed or endorsed by the publisher.

## Footnotes

1
http://pfam.janelia.org

2
http://www.ebi.ac.uk/interpro/search/sequence-search

3
http://web.expasy.org/compute_pi/

4
http://memesuite.org/tools/meme

5
http://smart.embl.de/

6
https://molbio.mgh.harvard.edu/sheenweb/main_page.html
