# Supplementary material for: Functional Characterization of Serotonin N-Acetyltransferase Genes (SNAT1/2) in Melatonin Biosynthesis of Hypericum perforatum
Source: Front Plant Sci. 2021 Dec 7;12:781717. doi: 10.3389/fpls.2021.781717 (PMC8688956; doi:10.3389/fpls.2021.781717)
Supplement: Supplementary file 1 [file Data_Sheet_1.ZIP › Raw Data/Raw Data.docx]

**Figure 1**

**(A/B)**The genome sequence data of *H. perforatum* are deposited in GenBank under project number PRJNA588586, and transcriptome sequence reads are deposited in the Sequence Read Archive (SRA) under accession number SRA: SRR8438983 (flowers), SRR8438984 (leaves), SRR8438985 (stems), and SRR8438986 (roots).

**(C)**The data of qPCR analysis of HpSNAT1 and HpSNAT2 in different tissues of *H. perforatum*, calculated with the equation $2^{-\Delta\Delta Ct}$ (F, flowers; L,leaves; R, roots; S, stems).

|  | F | L | R | S |
| --- | --- | --- | --- | --- |
| HpSNAT1 | 1.3205 | 5.6302 | 0.8827 | 1.5487 |
|  | 1.1846 | 7.1655 | 1.0140 | 2.0061 |
|  | 1.0554 | 7.6798 | 1.1173 | 1.0951 |
| HpSNAT2 | 1.2161 | 2.0499 | 0.9145 | 0.5137 |
|  | 1.0685 | 2.7970 | 1.2503 | 0.5792 |
|  | 1.1558 | 2.5286 | 1.3905 | 0.5766 |

**Figure 2**

**(A)**The original files (. oir) for subcellular localization of confocal microscope are attached.

**(B)**The data of qPCR analysis of HpSNAT1 and HpSNAT2 under drought (D-mannitol) and high-salt (NaCl) stress conditions. *H. perforatum*, calculated with the equation $2^{-\Delta\Delta Ct}$.

|  | 0h | 4h | 8h | 16h | 32h |
| --- | --- | --- | --- | --- | --- |
| HpSNAT1- D-mannitol | 0.8481 | 1.2431 | 2.3602 | 0.8023 | 0.3965 |
|  | 1.0250 | 1.0261 | 2.2354 | 0.8579 | 0.4608 |
|  | 1.1505 | 1.0108 | 2.5678 | 0.6623 | 0.4446 |
| HpSNAT1- NaCl | 0.7292 | 1.6169 | 2.6289 | 1.2844 | 1.7873 |
|  | 1.0726 | 2.1551 | 2.9372 | 1.6295 | 2.2675 |
|  | 1.2785 | 1.8503 | 3.1119 | 1.3545 | 2.3913 |
| HpSNAT2- D-mannitol | 0.8685 | 0.9223 | 0.9138 | 0.7186 | 0.8827 |
|  | 1.1070 | 1.1381 | 0.7071 | 0.7423 | 0.6492 |
|  | 1.0401 | 0.6537 | 1.2805 | 0.5340 | 0.6022 |
| HpSNAT2- NaCl | 0.9840 | 1.6769 | 1.1303 | 0.3399 | 1.4224 |
|  | 0.8409 | 0.7440 | 1.4540 | 0.8596 | 2.1386 |
|  | 1.2086 | 0.8606 | 1.2658 | 0.5446 | 1.9252 |

**Figure 3**

**(A)**Expression levels of HpSNAT in *snat*, Col-0, and five OE lines, analyzed by RT-PCR.

**
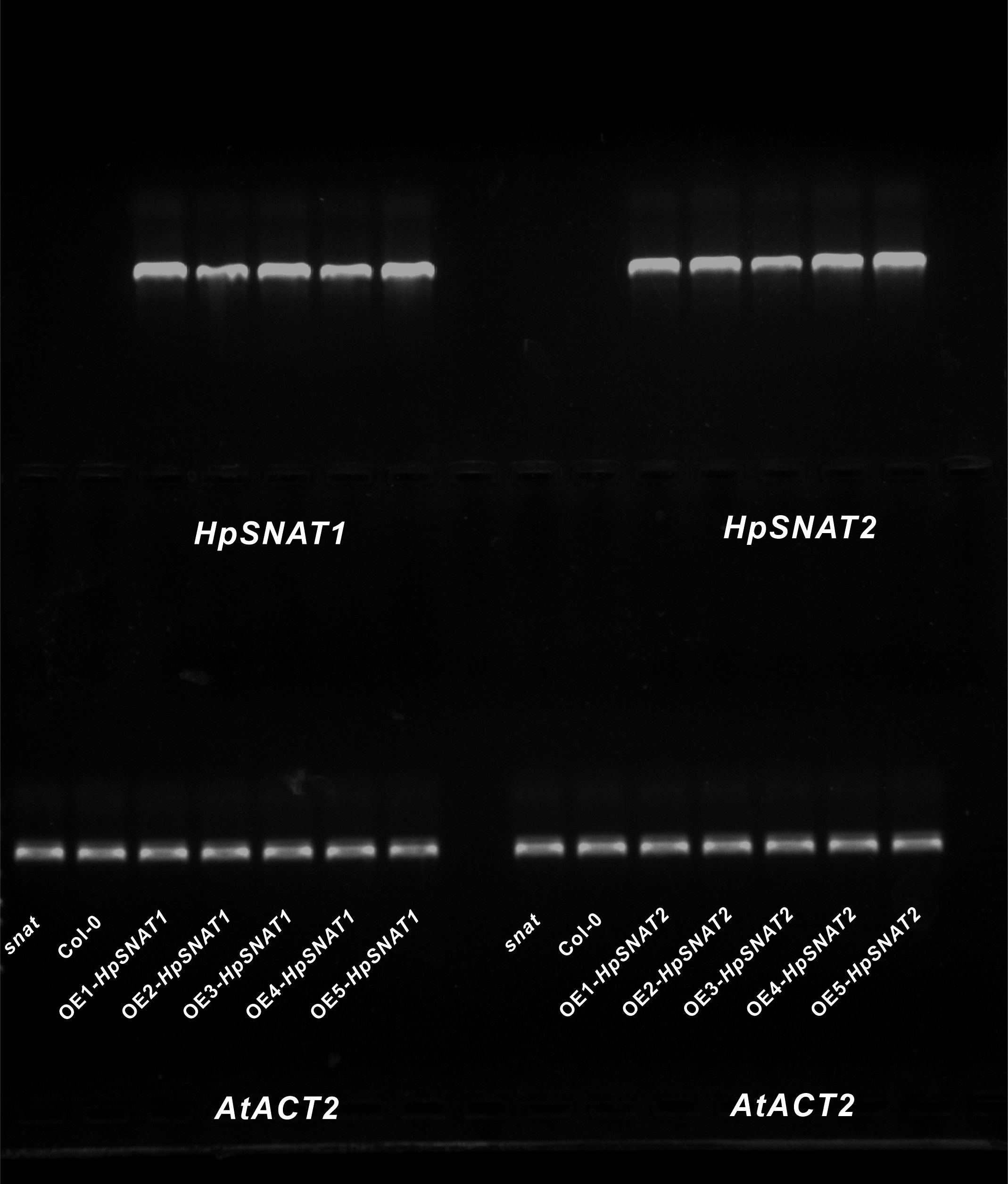
**

**(B)**Melatonin content in *snat*, Col-0, and five transgenic lines.

|  | **Melatonin Content (FW pg/g)** | | |
| --- | --- | --- | --- |
| *snat* | 34.38 | 32.58 | 35.08 |
| Col-0 | 37.61 | 43.22 | 40.96 |
| OE1-HpSNAT1 | 74.17 | 76.99 | 76.36 |
| OE2-HpSNAT1 | 66.31 | 67.25 | 66.55 |
| OE3-HpSNAT1 | 70.63 | 65.61 | 67.85 |
| OE4-HpSNAT1 | 62.05 | 60.29 | 61.53 |
| OE5-HpSNAT1 | 71.55 | 68.81 | 70.09 |
| OE1-HpSNAT2 | 60.98 | 58.23 | 60.73 |
| OE2-HpSNAT2 | 61.12 | 67.02 | 63.22 |
| OE3-HpSNAT2 | 62.41 | 58.36 | 60.02 |
| OE4-HpSNAT2 | 64.55 | 62.22 | 62.93 |
| OE5-HpSNAT2 | 70.02 | 69.99 | 63.07 |

**(C)**The fresh weight and dry weight of the rosette leaves in *snat*, Col-0, and OE seedlings.

|  | The Weight of Arabidopsis Plants (mg) | | | | | |
| --- | --- | --- | --- | --- | --- | --- |
|  | Fresh Weight | | | Dry Weight | | |
| *snat* | 125.63 | 121.95 | 119.66 | 10.36 | 11.59 | 11.02 |
| Col-0 | 177.26 | 170.23 | 182.36 | 15.36 | 15.92 | 18.25 |
| OE-HpSNAT1 | 230.82 | 239.66 | 227.92 | 24.56 | 25.97 | 22.96 |
| OE-HpSNAT2 | 302.55 | 320.98 | 317.5 | 27.85 | 24.69 | 24.05 |

**Figure 4**

**(A)**The primary root length and lateral root number of *snat*, Col-0, and OE seedlings under normal condition (control), D-mannitol, and NaCl treatments.

|  | The Primary Root Length (mm) | | | | | | | | |
| --- | --- | --- | --- | --- | --- | --- | --- | --- | --- |
|  | Control | | | D-mannitol | | | NaCl | | |
| *snat* | 67.2 | 68.5 | 67.1 | 66.2 | 67.6 | 65.3 | 61.3 | 63.4 | 59.8 |
| WT | 72.3 | 70.3 | 65.5 | 64.1 | 67.1 | 66.3 | 63.2 | 65.1 | 63.5 |
| OE-HpSNAT1 | 69.2 | 74.8 | 70.2 | 70.5 | 64.7 | 67.5 | 63.7 | 66.4 | 62.5 |
| OE-HpSNAT2 | 70.3 | 72.5 | 70.1 | 70.7 | 72.8 | 65.2 | 62.4 | 58.9 | 59.7 |
|  | The lateral root number | | | | | | | | |
|  | Control | | | D-mannitol | | | NaCl | | |
| *snat* | 12 | 14 | 13 | 19 | 20 | 22 | 10 | 12 | 13 |
| WT | 19 | 17 | 16 | 28 | 24 | 26 | 19 | 18 | 14 |
| OE-HpSNAT1 | 20 | 16 | 18 | 37 | 34 | 32 | 25 | 22 | 23 |
| OE-HpSNAT2 | 19 | 18 | 16 | 31 | 28 | 35 | 22 | 18 | 19 |

**(C)**The content of MDA and H_2_O_2_ in *snat*, Col-0, and OE lines under drought stress.

|  | MDA (nnmol/g) | | | H_2_O_2_(μmol/g) | | |
| --- | --- | --- | --- | --- | --- | --- |
| *snat* | 11.0217 | 13.8259 | 13.2926 | 3.4140 | 3.4726 | 3.5496 |
| WT | 10.1643 | 10.7830 | 11.3658 | 2.5667 | 2.6292 | 2.7854 |
| OE*-HpSNAT1* | 7.2663 | 6.6657 | 6.5896 | 1.6721 | 1.5885 | 1.8025 |
| OE-*HpSNAT2* | 6.8247 | 7.1067 | 7.3698 | 1.9926 | 1.6860 | 1.7025 |

**Figuge 5**

**(B)**Relative quantitative analysis of HpSNAT1 and HpSNAT2 expression in WT and OE hairy roots of *H. perforatum*, calculated with the equation $2^{-\Delta\Delta Ct}$.

| Lines | Reletive Expression Level (HpSNAT1) | | |
| --- | --- | --- | --- |
| WT | 1.1259 | 0.9124 | 0.9734 |
| OE-HpSNAT1-1 | 45.3595 | 47.8562 | 56.1028 |
| OE-HpSNAT1-2 | 65.7993 | 74.1993 | 83.4785 |
| OE-HpSNAT1-3 | 50.7968 | 35.4243 | 45.4644 |
| OE-HpSNAT1-4 | 62.9732 | 82.9019 | 64.8934 |
| OE-HpSNAT1-5 | 66.4102 | 68.7522 | 53.6932 |
|  | Reletive Expression Level (HpSNAT2) | | |
| OE-HpSNAT2-1 | 1712.8964 | 1409.6401 | 1270.4388 |
| OE-HpSNAT2-2 | 1352.2171 | 1345.983 | 1872.9687 |
| OE-HpSNAT2-3 | 1261.6632 | 1152.9475 | 1258.7515 |
| OE-HpSNAT2-4 | 1255.8465 | 1227.1629 | 1641.8551 |
| OE-HpSNAT2-5 | 1230.0016 | 1118.8321 | 1282.2345 |

**(D)**Relative quantitative analysis of HpSNAT1 and HpSNAT2 expression in WT and OE leaves of *H. perforatum*, calculated with the equation $2^{-\Delta\Delta Ct}$.

| Lines | Reletive Expression Level (HpSNAT1) | | |
| --- | --- | --- | --- |
| WT | 0.9578 | 0.9893 | 0.9019 |
| OE-HpSNAT1-2 | 19.9425 | 20.9824 | 21.4729 |
| OE-HpSNAT1-4 | 25.9521 | 22.5405 | 27.3053 |
| OE-HpSNAT1-5 | 28.203 | 22.5926 | 29.4007 |
|  | Reletive Expression Level (HpSNAT2) | | |
| OE-HpSNAT2-1 | 371.6441 | 353.2252 | 376.832 |
| OE-HpSNAT2-2 | 133.5384 | 137.9286 | 109.2214 |
| OE-HpSNAT2-4 | 234.6627 | 210.029 | 325.0335 |

**Figure 6**

**(A)**The content of 5-HT, 5-MT, NAS, and melatonin in WT and OE leaves of *H. perforatum* plants.

|  | 5-HT (μg/g, DW) | | | N-ACE (mg/g, DW) | | |
| --- | --- | --- | --- | --- | --- | --- |
| EV | 8.6977 | 8.4730 | 7.8517 | 1.5293 | 2.0996 | 1.9287 |
| OE-HpSNAT1-2 | 19.5765 | 22.7753 | 18.2943 | 4.2052 | 3.4162 | 3.8001 |
| OE-HpSNAT1-4 | 20.5811 | 16.3776 | 20.6340 | 5.0019 | 5.2399 | 5.5698 |
| OE-HpSNAT1-5 |  | 18.0960 | 20.9512 | 3.0231 | 4.0191 | 3.1164 |
| OE-HpSNAT2-1 | 20.3432 | 27.5208 | 28.4989 | 2.8658 | 3.1103 | 3.4156 |
| OE-HpSNAT2-2 | 20.3684 | 18.2150 | 19.3121 | 2.8993 |  | 3.5229 |
| OE-HpSNAT2-4 | 17.3690 | 21.1213 | 20.3696 | 2.0534 | 2.2111 | 2.5192 |
|  | 5-MT (μg/g, DW) | | | Melatonin (μg/g, DW) | | |
| EV | 90.6660 | 84.2783 | 125.6913 | 4.1762 | 4.6621 | 4.6207 |
| OE-HpSNAT1-2 | 249.3644 | 211.3313 | 210.4139 | 8.2904 | 9.4740 | 11.6345 |
| OE-HpSNAT1-4 | 287.2598 | 283.1250 | 256.3563 | 20.4263 | 17.3045 | 15.5806 |
| OE-HpSNAT1-5 | 188.1274 | 182.0660 | 123.4371 | 21.0517 | 18.4932 | 23.3000 |
| OE-HpSNAT2-1 | 410.4217 | 396.2413 | 390.5691 | 24.5250 | 20.4697 | 18.4571 |
| OE-HpSNAT2-2 | 313.2683 | 331.0293 | 326.5543 | 16.6067 | 17.9619 | 18.9955 |
| OE-HpSNAT2-4 | 263.4995 | 291.5842 | 315.8895 | 10.8307 | 13.7847 | 14.9734 |

**(B)**

|  | HpASMT1 | | | HpASMT3 | | |
| --- | --- | --- | --- | --- | --- | --- |
| EV | 0.9124 | 0.8692 | 0.9188 | 0.7797 | 0.7810 | 1.1980 |
| OE-HpSNAT1-2 | 2.0015 | 2.1108 | 1.8762 | 3.5884 | 3.0667 | 3.0667 |
| OE-HpSNAT1-4 | 2.5102 | 2.1344 | 2.4079 | 2.7116 | 2.6268 | 2.9828 |
| OE-HpSNAT1-5 | 1.9423 | 2.0134 | 1.8618 | 2.6697 | 2.5295 | 2.8945 |
| OE-HpSNAT2-2 | 0.8054 | 0.8793 | 0.7816 | 1.5340 | 1.3787 | 1.5980 |
| OE-HpSNAT2-1 | 1.1104 | 0.8793 | 0.8895 | 1.7000 | 1.3851 | 1.5619 |
| OE-HpSNAT2-4 | 0.9040 | 0.8148 | 0.8854 | 1.5619 | 1.9000 | 1.4675 |
