# Supplementary figures and images for: Functional Characterization of Serotonin N-Acetyltransferase Genes (SNAT1/2) in Melatonin Biosynthesis of Hypericum perforatum
Source: Front Plant Sci. 2021 Dec 7;12:781717. doi: 10.3389/fpls.2021.781717 (PMC8688956; doi:10.3389/fpls.2021.781717)

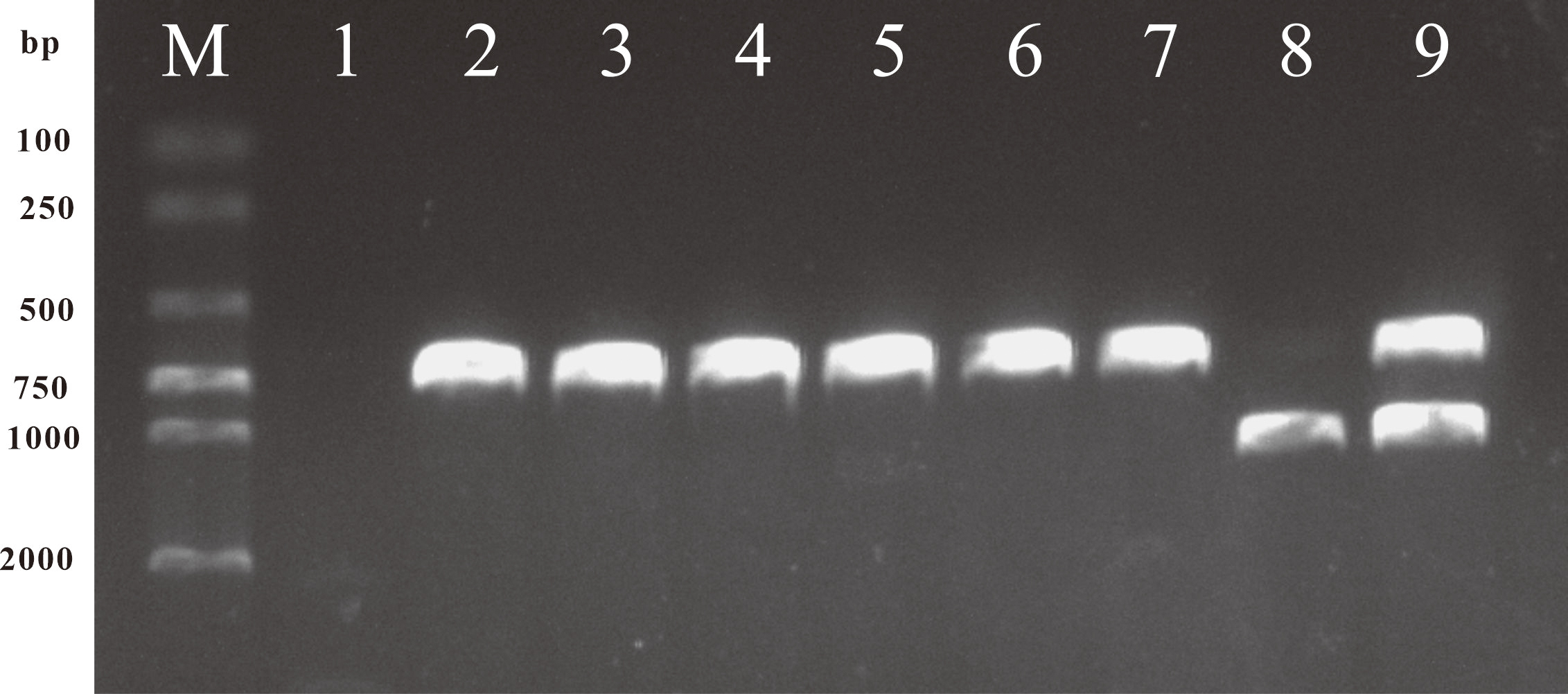

Supplement: Supplementary file 2 [file Image_1.JPEG]

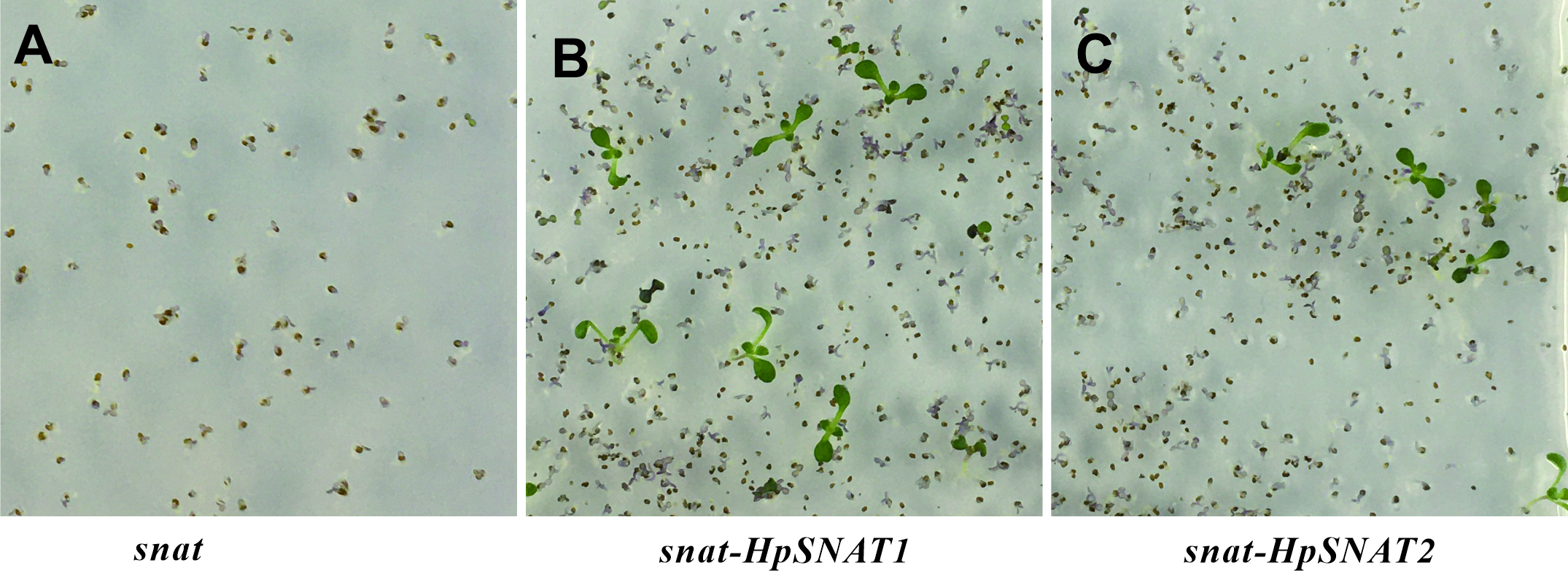

Supplement: Supplementary file 3 [file Image_2.JPEG]

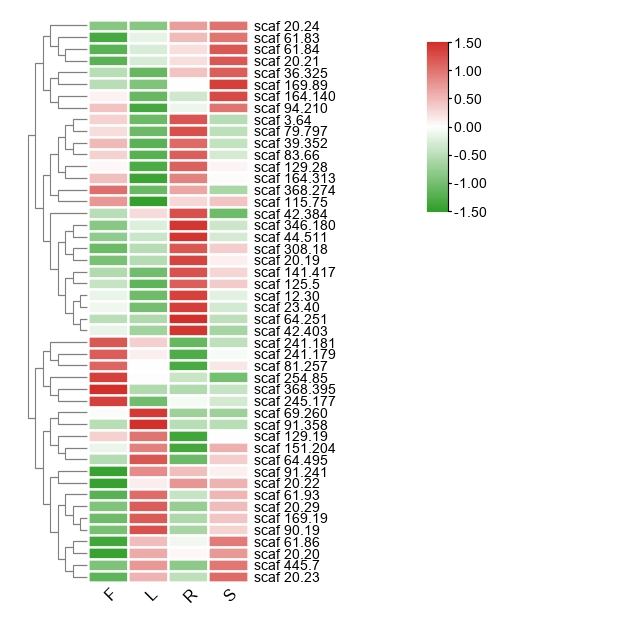

Supplement: Supplementary file 4 [file Image_3.JPEG]
